# The Role and Mechanism of *Paeoniae Radix Alba* in Tumor Therapy

**DOI:** 10.3390/molecules29071424

**Published:** 2024-03-22

**Authors:** Yating Yang, Ling Yuan, Kaili Wang, Doudou Lu, Fandi Meng, Duojie Xu, Weiqiang Li, Yi Nan

**Affiliations:** 1 Traditional Chinese Medicine College, Ningxia Medical University, Yinchuan 750004, China; 2 College of Pharmacy, Ningxia Medical University, Yinchuan 750004, China; 3 School of Clinical Medicine, Ningxia Medical University, Yinchuan 750004, China; 4 Key Laboratory of Ningxia Minority Medicine Modernization, Ministry of Education, Ningxia Medical University, Yinchuan 750004, China; 5 Department of Chinese Medical Gastrointestinal, The Affiliated TCM Hospital, Ningxia Medical University, Wuzhong 751100, China

**Keywords:** *Paeoniae Radix Alba*, tumor therapy, medicine and food homology, molecular mechanism

## Abstract

Tumors have a huge impact on human life and are now the main cause of disease-related deaths. The main means of treatment are surgery and radiotherapy, but they are more damaging to the organism and have a poor postoperative prognosis. Therefore, we urgently need safe and effective drugs to treat tumors. In recent years, Chinese herbal medicines have been widely used in tumor therapy as complementary and alternative therapies. Medicinal and edible herbs are popular and have become a hot topic of research, which not only have excellent pharmacological effects and activities, but also have almost no side effects. Therefore, as a typical medicine and food homology, some components of *Paeoniae Radix Alba* (PRA, called Baishao in China) have been shown to have good efficacy and safety against cancer. Numerous studies have also shown that *Paeoniae Radix Alba* and its active ingredients treat cancer through various pathways and are also one of the important components of many antitumor herbal compound formulas. In this paper, we reviewed the literature on the intervention of *Paeoniae Radix Alba* in tumors and its mechanism of action in recent years and found that there is a large amount of literature on its effect on total glucosides of paeony (TGP) and paeoniflorin (PF), as well as an in-depth discussion of the mechanism of action of *Paeoniae Radix Alba* and its main constituents, with a view to promote the clinical development and application of *Paeoniae Radix Alba* in the field of antitumor management.

## 1. Introduction

Cancer continues to grow globally and has become the second leading cause of death worldwide [1,2]. At present, the main treatment modalities are surgical resection, radiotherapy and chemotherapy [3], but these treatments are more damaging to the organism, and the treatment process is characterized by high toxicity and side effects, a poor postoperative prognosis and a high recurrence rate [4,5]. The quality of patients’ survival is still not effectively ensured, and their metastasis and recurrence cannot be prevented [6,7]. Therefore, there is an urgent need to find non-toxic natural drugs to treat tumors [8].

With the prosperous development of Chinese medicine, Chinese herbal medicines have been widely used in tumor treatment as complementary and alternative therapies [9]. They have the advantages of good therapeutic effects and low toxicity and side effects, which can ensure the quality of survival and prolong the survival rate of cancer patients [10,11]. In recent years, more and more people have started to pay attention to Chinese herbs with health benefits and therapeutic effects [12]. Most of the Chinese herbs can be used for both healing and dietary purposes, so they are called medicinal herbs. The concept of medicine and food homology has been around for a long time, dating back to the “*Huangdi Neijing*” [13], where it is mentioned that food can be used as medicine to enhance one’s immunity and thus prevent or treat diseases [14,15]. With the change in time, the theory of the medicine and food homology has been enriched and developed, and its core content has continued to this day. *Paeoniae Radix Alba* is one of these herbs, and as a natural medicine, it has been used for nearly a thousand years.

*Paeoniae Radix Alba* (PRA) is the dried root of *Paeonia lactiflora Pall*. in the buttercup family [16]. PRA has a wide range of uses and has been widely used clinically for the treatment of visceral pain [17,18], cancerous pain [19], chronic gastritis [20,21], chronic hepatitis B [22], rheumatoid arthritis, etc. [23]. It also has a high frequency of use in the prevention and treatment of tumors [24]. With the in-depth development of chemical technology, there have been many reports on the pharmacological effects and mechanisms of PRA and its active ingredients, confirming that it has a wide range of pharmacological effects, including analgesic [25], anti-inflammatory [26], antidepressant [27,28], hepatoprotective [29], immune modulation [30] and significant antitumor activity, and it plays a therapeutic role in gastric cancer, hepatocellular carcinoma, pancreatic cancer and other malignant tumors [31,32]. This paper mainly introduces the main active ingredients of PRA and summarizes the mechanism of action of PRA and its active ingredients in the treatment of various malignant tumors.

## 2. Effects of the Main Active Ingredients of PRA

The chemical composition of PRA is diverse, mainly including monoterpenes and their glycosides, triterpenoids, flavonoids, tannins and polysaccharides [33,34]. Among them, the ones with significant efficacy and wide application in the treatment of various tumors are total glucosides of paeony (TGP) and paeoniflorin (PF). TGP is considered to be the main bioactive component of PRA, while PF is the main component of TGP [35]. The chemical structure formula is shown in Table 1.

### 2.1. Total Glucosides of Paeony (TGP)

Total glucosides of paeony (TGP) is a group of glycosides in the Chinese herb PRA [36], including paeoniflorin, paeonin [37], albiflorin [38,39], hydroxy-paeoniflorin [40], benzoyl-paeoniflorin [41], etc. [42,43]. Modern pharmacological studies have found that TGP not only has immunomodulatory, anti-inflammatory and hepatoprotective effects, but also has a variety of effects such as inhibition of cell proliferation [44,45], which has good prospects for application and development.

### 2.2. Paeoniflorin (PF)

Paeoniflorin (PF), the main active ingredient of PRA, is a naturally occurring pharmacologically active ingredient that is highly valued for its low toxicity, high efficiency and safety, as well as for its association with a variety of antitumor, inflammatory, depressive and oxidative stress pharmacological effects [46,47]. In recent years, scholars at home and abroad have conducted in-depth studies on the antitumor effects of PF and found that PF has a good inhibitory effect for hepatocellular carcinoma, gastric carcinoma, intestinal carcinoma, lung carcinoma, leukemia [48] as well as skin carcinoma [49], and its mechanism of action is related to apoptosis induced by different signaling pathways, preventing the proliferation of cancer cells and inhibiting metastasis [50,51].

### 2.3. Others

In addition to TGP and PF, PRA contains various active ingredients such as albiflorin, oxypaeoniflorin, 6′-O-galloylpaeoniflorin and paeoniflorigenone [52]. However, TGP and PF are more well-studied for tumor therapeutic purposes, and therefore, the focus of this paper is on TGP and PF.

## 3. Antitumor Therapy Mechanisms

Inhibition of tumor cell proliferation, induction of apoptosis, inhibition of cell metastasis, anti-inflammation and immunomodulation are the main pathways and phenotypes of the antitumor effects of PRA [53,54], as shown in Table 2.

### 3.1. Inhibits Tumor Cell Proliferation

The most basic characteristics of a cancer cell include its ability to proliferate persistently and over a long period of time [55]. The coordinated action between out-of-control cell metabolism, growth and proliferation is essential for tumorigenesis [56]. Cancer cells interfere with normal signals in the body that autonomously control cell growth [57,58]. These signaling pathways are able to regulate the entire process through the cell cycle and cell growth, also known as cell size increase. PF derived from PRA was found to be a potential novel therapeutic agent for gastric cancer. PF acted on gastric cancer MGC803 cells and could inhibit cell viability and induced apoptosis by up-regulating miR-124 and inhibiting PI3K/Akt and STAT3 signaling [59]. PF also inhibited the proliferation and invasion of breast cancer cells in the Notch-1 signaling pathway by interfering with breast cancer MDA-MB-231 and MCF-7 cells [60]. In addition, TGP extracted from PRA inhibited the proliferation, invasion and migration ability of laryngeal cancer Hep-2 cells in a concentration-dependent manner, a process that may be related to the inhibition of the activation of the PI3K/Akt/GSK3β signaling pathway [61]. Thus, PF and TGP can inhibit cancer cell proliferation through signaling pathway regulation.

### 3.2. Induces Apoptosis in Tumor Cells

Apoptosis is a fundamental biological phenomenon of the cell and is a tightly controlled polygenic process [62]. If there is a problem with this process, apoptosis does not occur in cells that are supposed to die, and damaged cells can survive and develop into cancer cells [63,64]. In the case of cancer cells, an interruption of the apoptotic process means the development and spread of cancer [65,66]. Therefore, balancing the process of cellular metabolism in the body can combat the onset and development of cancer [67]. It was found that PF intervention in gastric cancer SGC7901 cells significantly inhibited NF-kappaB activity and enhanced 5-fluorouracil-induced apoptosis in gastric cancer cells by preventing I kappaB alpha phosphorylation and reducing the nuclear translocation of NF-kappaB [68]. Further, apoptosis was significantly induced in human cervical cancer cell line HeLa cells after PF treatment, which could result in the down-regulation of the anti-apoptotic gene Bcl-2 and the up-regulation of the pro-apoptotic genes Bax and caspase-3 [69]. In addition, PF enhances the expression of caspase-9 and -3 proteins as well as Bax proteins in prolactinoma MMQ cells, whereas it inhibits the expression of Bcl-2 proteins and induces apoptosis mediated by the mitochondrial pathway [70]. The apoptosis-inducing effect is seen in a variety of cancers.

### 3.3. Inhibits Tumor Metastasis

Malignant tumor cells have the ability to invade surrounding tissues and metastasize to distant tissues [71], and in some cases, they can penetrate the body’s barriers and destroy surrounding tissues after invading the environment [72,73]. Once the cancer metastasizes, it is difficult to control and will spread step-by-step in the body. Secondly, early tumors are difficult to detect, and by the time obvious symptoms appear, the tumor is likely to be in the middle or late stage of malignant transformation, so the process of controlling metastasis is particularly urgent [74]. It was found that PF acted on gastric cancer AGS cells and inhibited the migration and invasion-promoting ability of gastric cancer-associated fibroblasts (GCAFs) by targeting microRNA-149 and IL-6 [75]. PF also decreased the expression of MMP-9 and ERK in hepatocellular carcinoma HepG2 and Bel-7402 cells and increased the expression of E-cadherin in both cell lines [76]. PF also significantly decreased the expression of Vimentin in colorectal cancer cells while increasing the expression of E-cadherin, which in turn reversed the EMT process [77,78]. It can be seen that PF can inhibit metastasis by regulating matrix metalloproteinases and EMT-related proteins [79,80]. In addition, TGP could inhibit the metastasis of pancreatic cancer ASPC-1 cells, and the expression levels of MMP-2 and MMP-9 proteins in the cells of the TGP group were significantly reduced compared with those of the blank control group [81]. Therefore, both PF and TGP are involved in regulating the anticancer microenvironment [82], which inhibits the growth of cancer cells while suppressing their metastasis.

### 3.4. Anti-Inflammatory and Immunomodulatory

Inflammation has been shown to be closely associated with the stage of development of action and malignant progression of most types of tumors, as well as with the efficacy of anticancer therapies [83,84]. Specifically, long-term chronic inflammation is associated with immunosuppression and would provide a favorable microenvironment for tumorigenesis, progression and metastasis [85,86]. Therefore, anti-inflammation and immunomodulation have unique advantages in regulating the tumor microenvironment, and immunotherapy is becoming more and more important in the treatment of tumors [87,88]. PRA, as a kind of traditional Chinese medicine, overcomes the characteristics of western medicines, which have high side effects and lower immunity, and has a natural advantage in anti-inflammation and immunomodulation therapy [89,90,91]. In a mouse model, PF improved survival and reduced the number and size of colon tumors. Medicinal plants and their extracts with anti-inflammatory and immunomodulatory properties may be an effective strategy for the treatment and prevention of chronic inflammation in colitis-associated colorectal cancer by mechanisms that may be related to the inhibition of the IL-6/STAT3 signaling pathway and IL-17 levels [92,93]. Controlling inflammation will open new possibilities for long-term, multilevel tumor control [94].

**Table 2:** The therapeutic mechanism of *Paeoniae Radix Alba* and its active ingredients in antitumor therapy.

Cancer	Ingredient	Experimental Model	Mechanism	Target of Action	Phenotype	Ref.
Gastric cancer	Paeoniflorin	AGS	IL-6-STAT3-MMP	Inhibition of IL-6 production and secretion by up-regulation of microRNA149 expression in GCAFs and subsequent blocking of IL-6-STAT3-MMP signaling in AGS cells activated by GCAFs	Metastasis	[75]
Gastric cancer	Paeoniflorin	MGC803, SGC7901	Hippo	Inhibits cell growth, enhances apoptosis and reduces cell infiltration	Proliferation, apoptosis, metastasis	[95]
Gastric cancer	Paeoniflorin	MGC803	microRNA-124, PI3K/AKT/STAT3	Inhibits cell activity and induces apoptosis by up-regulating miR-124 and inhibiting PI3K/Akt and STAT3 signaling	Proliferation apoptosis	[59]
Gastric cancer	Paeoniflorin	SGC7901	NF-κB	Inhibits NF-κB activity and enhances apoptosis in gastric cancer cells	Apoptosis	[68]
Breast cancer	Paeoniflorin	MDA-MB-231, MCF-7	Notch-1	PF inhibits the proliferation and invasion of breast cancer cells by inhibiting the Notch-1 signaling pathway	Proliferation, metastasis	[60]
Breast cancer	Paeoniflorin	MCF-7	miR-15b/FOXO1/CCND1/β-catenin	Inhibition of breast cancer cell growth, pro-apoptosis and promotion of FOXO1 expression through down-regulation of miR-15b, leading to transcriptional inhibition of CCND1 and subsequent blockade of β-catenin protein signaling	Proliferation, apoptosis	[96]
Pancreatic cancer	Paeoniflorin	Panc-1	Mitochondrial apoptosis pathway	The expression of caspase-3 and Cleave caspase3 increased with the increase in PF concentration, and the opposite was true for Bcl-2	Proliferation, apoptosis	[97]
Pancreatic cancer	Paeoniflorin	Capan-1, MIAPaCa-2	HTRA3	Decreased cell proliferation and increased apoptotic Bax protein expression	Proliferation, apoptosis	[98]
Pancreatic cancer	Paeoniflorin	BxPC-3 and L3.6pl. A tumor model in BALB/c nude mice	ErbB3/PI3K/AKT	Inhibition of ErbB3/PI3K/Akt phosphorylation	Proliferation, apoptosis	[99]
Pancreatic cancer	Total glucosides of paeony	ASPC-1	-	PCNA, MMP-2, MMP-9 protein and mRNA expression levels were significantly reduced in the administered group	Proliferation, metastasis	[81]
Tongue cancer	Total glucosides of paeony	HSC3	LINC00319/miR-608	LINC00319 targets to negatively regulate miR-608 expression and LINC00319 overexpression reverses the effect of TGP on proliferation, migration and invasion of HSC3 cells	Proliferation, metastasis	[100]
Laryngeal cancer	Total glucosides of paeony	Hep-2	PI3K/Akt/GSK3β	p-PI3K, p-Akt, p-GSK3β protein expression levels were significantly reduced	Proliferation, metastasis	[61]
Liver cancer	Paeoniflorin	BEL-7402	Hedgehog/Gli	Associated with inhibition of Hedgehog/Gli signaling pathway activation, inhibition of MAPK/ERK pathway activity and inhibition of MMP-9 expression	Metastasis	[101]
Liver cancer	Paeoniflorin	HepG2, BEL-7402	MMP-9/E-CAD/ERK	Growth inhibition and significant reduction in invasion, metastasis and adhesion of hepatocellular carcinoma cell lines	Metastasis	[76]
Liver cancer	Paeoniflorin	HepG2, SMMC-7721	Mitochondrial apoptosis pathway	Induction of apoptosis in hepatocellular carcinoma cells through down-regulation of EP2 expression and concomitant increase in Bax/Bcl-2 ratio, leading to up-regulation of caspase-3 activity	Proliferation, apoptosis	[102]
Liver cancer	Paeoniflorin	HepG2, SMMC-7721	5-HT1D, Wnt/β-catenin	Blocking Wnt/β-conjugated protein pathway expression by down-regulating 5-HT1D	Proliferation, metastasis	[103]
Lung cancer	Paeoniflorin	A tumor model in C57BL/6J mice	-	Lung metastatic colonization in Lewis lung cancer-loaded mice in the paeoniflorin group was significantly less than that in the model group	Metastasis	[104]
Lung cancer	Paeoniflorin	A549	Fas/APO-1	Antiproliferative activity is mediated by cell cycle arrest in G0/G1 phase block and Fas/Fas ligand-mediated apoptotic pathways	Proliferation, apoptosis	[105]
Ovarian cancer	Paeoniflorin	HO8910	Mitochondrial apoptosis pathway	The expression level of intracellular caspase-3 in the paeoniflorin-treated group was significantly higher than that in the control group, and the expression levels of Bcl-2 and nuclear factor-κB p56 were significantly lower than those in the control group	Proliferation, apoptosis, metastasis	[106]
Cervical carcinoma	Paeoniflorin	HeLa	Mitochondrial apoptosis pathway	Decreased expression of Bcl-2 and enhanced expression of Bax and caspase-3	Apoptosis	[69]
Colorectal cancer	Paeoniflorin	A tumor model in CAC mice	IL-6/STAT3	PF increased survival and decreased the number and size of colon tumors in mice	Anti-inflammatory, immunomodulation	[92]
Colorectal cancer	Paeoniflorin	A tumor model in CAC mice. HT29	p53/14-3-3	Cell cycle arrest mainly in G1 phase, activation of caspase-3 and caspase-9 demonstrated the pro-apoptotic effect of PF	Proliferation, apoptosis	[107]
Colorectal cancer	Paeoniflorin	HCT116	FOXM1	PF inhibited cell growth and induced apoptosis and suppressed cell cycle progression in the G0/G1 phase. It also inhibited colorectal cancer cell migration and invasion	Proliferation, metastasis	[108]
Colorectal cancer	Paeoniflorin	HCT116, SW480	EMT	Inhibition of migration and invasive ability of colorectal cancer cells and reversal of epithelial–mesenchymal transition by suppressing HDAC2 expression	Metastasis	[77]
Multiple myeloma	Paeoniflorin	SKO-007	MMP-2/microRNA(miR)-29b	Inhibition of cell proliferation and promotion of apoptosis in multiple myeloma cells by inhibiting MMP-2 expression in multiple myeloma cells through miR-29b up-regulation	Proliferation, apoptosis	[109]
Osteosarcoma	Paeoniflorin	HOS, Saos-2	Mitochondrial apoptosis pathway	G2/M phase cell cycle arrest and apoptosis	Proliferation, apoptosis	[110]
Glioma	Paeoniflorin	U87	MMP-9/microRNA-16	Increased miR-16 expression and decreased MMP-9 protein expression	Proliferation, apoptosis	[111]
Glioma	Paeoniflorin	U87, U251	Ubiquitin-proteasome pathway	Proteasome-dependent STAT3 degradation induces growth inhibition and apoptosis in human glioma cells	Proliferation, apoptosis	[112]
Glioblastoma	Paeoniflorin	T98G, U251	HGF/c-Met/RhoA/ROCK	Inhibition of HGF/c-Met/RhoA/ROCK signaling pathway	Metastasis	[113]
Glioblastoma	Paeoniflorin	U87, U251 and T98G cells. A tumor model in BALB/c nude mice	EMT, TGFβ/MMP-2/9	Treatment of glioblastoma by inhibition of the TGFβ signaling pathway and inhibition of EMT	Metastasis	[78]
Prolactinoma	Paeoniflorin	MMQ, GH3	Mitochondrial apoptosis pathway	Inhibition of proliferation and induction of apoptosis in prolactinoma cells	Proliferation, apoptosis	[70]
Bladder carcinoma	Paeoniflorin	RT4. A tumor model in mice	Mitochondrial apoptosis pathway	Pro-apoptotic, blocked the translocation of STAT3 to the nucleus	Proliferation, apoptosis	[114]

**Figure 1:**
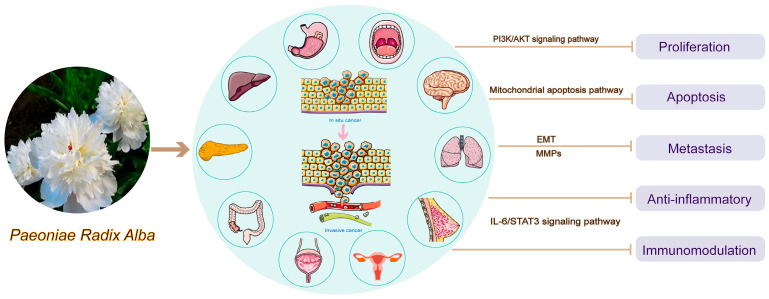
Antitumor mechanism of *Paeoniae Radix Alba*.

In general, PRA and its active ingredients or derivatives mainly produce antitumor effects on cell proliferation, apoptosis and metastasis and anti-inflammation and immunomodulation through signaling pathways, mitochondrial apoptotic pathway, EMT, MMPs, etc., as shown in Figure 1.

## 4. Preventive Effect

PRA is found in many foods and medicines and not only has antitumor activity, but also has auxiliary therapeutic and preventive effects in many diseases [115]. Postoperative patients with low immunity can take soup with PRA to improve their physical condition and enhance their immunity [116,117]. In the late stage of various diseases, it helps patients having a loss of appetite to open the stomach and replenish nutrition in time [118]. Long-term illnesses, bedridden conditions, all kinds of chronic diseases and the patients’ long-term medication will damage the spleen and stomach, and a medicinal diet made of PRA can be very good to protect the patient’s spleen and stomach, without harming the organism, but also to achieve the role of treatment of the disease [119,120]. Therefore, we can consume medicinal meals with PRA for a long time to regulate the body. As shown in Table 3, we add PRA to many nutritious porridges, medicinal diets and other foods, which play a key role in preventing diseases.

## 5. Conclusions and Outlook

The current treatment of tumors is still conventional Western medicine, which can quickly kill tumor cells and reduce the tumor load, but it also inevitably brings a series of toxic side effects and adverse reactions [130]. Traditional Chinese medicine and its long-term clinical experience have shown that PRA has a tonic effect and has therapeutic and preventive effects on a variety of diseases [131]. Among them, it has a remarkable pain-relieving effect, which can significantly relieve cancer pain and alleviate patients’ suffering. Modern pharmacological studies have shown that PRA has a wide range of anticancer activity, with obvious inhibitory effect on tumor cells, which can reduce the invasion and migration of cancer cells and inhibit the spread of cancer cells. In addition, PRA can enhance human immunity, making cancer patients more resistant to cancer cells. The concept of medicine and food homology is based on the advantage of boosting one’s immunity, thus achieving the effect of preventing and treating diseases [132,133]. PRA has a long history of medicinal use, and as a dual-purpose herb, it has great development and application value in clinical application, health food and diet [134].

We searched PubMed, Google Scholar, and China national knowledge infrastructure (CNKI) with the keywords “*Paeoniae Radix Alba*”, “cancer”, “carcinoma”, “tumor” and so on and found that there are no reviews on the treatment of tumors by PRA in the past five years. More attention was paid to PRA and its active ingredients for the treatment of single cancers such as breast cancer and colorectal cancer. We retrieved a review of paeoniflorin for tumor therapy published in 2022 [135], which focused only on paeoniflorin’s tumor-suppressing effects through various mechanisms and modulation of signaling pathways, but it talked little about paeoniflorin derived from the traditional Chinese medicine PRA, which is of little reference value to the advantages of traditional Chinese medicine.

In summary, this paper reviewed the application of PRA and its active ingredients, TGP and PF, in tumor therapy, focusing on the antitumor mechanism of action, medicinal food and treatment of disease and other advantages of PRA, which can inhibit the occurrence and malignant progression of tumors.

## Figures and Tables

**Table 1:** Active ingredients and structural formula of *Paeoniae Radix Alba*.

Name	Structural Formula
Total glucosides of paeony	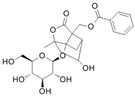
Paeoniflorin	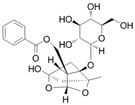

**Table 3:** *Paeoniae Radix Alba* diet prescription.

Name	Ingredient	Methods of Production	Efficacy	Book
Baishao decoction	*Paeoniae Radix Alba* (Baishao) 10 g, rice 50 g	*Paeoniae Radix Alba* thick decoction of the juice added to boiled rice into thin porridge to serve, 1 dose per day	Nourish the Yin and soften the liver	*Watching Red Mansions and Talking about Chinese Medicine* [121]
Baishao Fushen decoction	*Paeoniae Radix Alba* (Baishao), *Sophora flavescens Aiton* (fushen) each 10 g, rice 100 g, moderate amount of icing sugar	All the medicines’ decocted juices added to rice porridge, to be cooked with rock sugar and then boiled, 1 dose per day	Clearing the liver and laxing fire, submerging Yang and tranquilizing the mind	*Watching Red Mansions and Talking about Chinese Medicine*
Shanyao Baishao decoction	*Dioscorea oppositifolia* L. (Shanyao) 50 g, *Paeoniae Radix Alba* (Baishao) 15 g, rice 100 g, moderate amount of icing sugar	The two medicines’s decocted juice added to rice porridge, to be cooked with rock sugar and then boiled, 1 dose per day	Strengthens the spleen and softens the liver	*Watching Red Mansions and Talking about Chinese Medicine*
Shiquan Dabu decoction	*Angelica sinensis (Oliv.) Diels* (Danggui), *Paeoniae Radix Alba* (Baishao), *Codonopsis pilosula (Franch.) Nannf.* (Dangshen), *Smilax glabra Roxb.* (Fuling), *Rehmannia glutinosa (Gaertn.) DC.* (Shudihuang), *Atractylodes macrocephala Koidz.* (Baizhu) each 10 g, *Conioselinum anthriscoides ‘Chuanxiong’* (Chuanxiong) 8 g, *Astragalus mongholicus Bunge* (Huangqi) 5 g, *Neolitsea cassia* (L.) *Kosterm.* (Rougui) 4 g, *Glycyrrhiza glabra* L. (gancao) 5 g, *Zingiber officinale Roscoe* (Shengjiang) 25 g, 1000 g of pork elbow, 500 g of raw chicken bones	Put the first 10 flavors of medicine into a sand jar with water and decoct twice. Wash the hairs of the pork elbow, put it on a high fire until slightly charred and put it into the rice water to soak for 30 min. Fish and chicken bones added together into a casserole, add fresh soup to boil, simmer over medium heat for 90 min and then simmer over low heat until the elbow meat is soft and rotten.	Nourishing the liver and kidney, nourishing the heart and spleen, benefiting the vital energy and blood, tonifying the essence, and stimulating the brain and intellect	*Taiping Huimin Hekebao Formula* [122]
Shanyao Gancao decoction	*Paeoniae Radix Alba* (Baishao) 30 g, *Glycyrrhiza glabra* L. (Gancao) 10 g, rice 50 g	First decoction of *Paeoniae Radix Alba*, licorice juice to remove slag, add round-grained rice to cook into thin rice and served warm in the morning and evening	Stops pain	*Tumor Food Therapy Expert Talk* [123]
Guishen Niurou decoction	*Angelica sinensis (Oliv.) Diels* (Danggui), *Codonopsis pilosula (Franch.) Nannf.* (Dangshen), *Conioselinum anthriscoides ‘Chuanxiong’* (Chuanxiong), *Paeoniae Radix Alba* (Baishao) each 10 g, *Morus alba* L. (Sangzhi), *Hansenia weberbaueriana (Fedde ex H.Wolff) Pimenov and Kljuykov* (qianghuo) each 15 g, *Glycyrrhiza glabra* L. (gancao) 5 g, 500 g of lamb	Cut the mutton and *Paeoniae Radix Alba* into pieces, wrap them in a medicinal cloth, add water and simmer them together until the mutton is cooked; cook and eat 2 times a day	Promoting blood circulation and removing blood stasis, clearing channels and relieving pain	*Chinese Medicine Rehabilitation Terminology* [124]
Jianpi Xiaopi soup	*Atractylodes macrocephala Koidz.* (Baizhu), *Paeoniae Radix Alba* (Baishao), *Citrus aurantium* L. (Zhiqiao), rice	Put *Paeoniae Radix Alba* and corn into a casserole dish, add appropriate amount of water, bring to a boil over high heat, then switch to low heat and simmer for 30 min	Suppressing the liver and harmonizing the spleen, promoting qi circulation, eliminating lumps and relieving pain	*Diagnosis and treatment of gastric diseases* [125]
Fufang Huangqi decoction	*Astragalus mongholicus Bunge* (Huangqi) 15 g, 10 g each of *Paeoniae Radix Alba* (Baishao) and *Neolitsea cassia* (L.) *Kosterm.* (Guizhi), *Zingiber officinale Roscoe* (Shengjiang) 15 g, *Ziziphus jujuba Mill.* (Dazao) 4, rice 100 g	Wash clean round-grained rice and jujube and cook into thin porridge	Benefiting qi and nourishing blood, warming the meridians and clearing the channels	*Specialized Chinese Medicine Treatment for Stroke* [126]
Baishao Huanyu decoction	*Paeoniae Radix Alba* (Baishao) 20 g, 1 grass carp (about 1000 g)	Grass carp slaughtered and washed offal, fish body on both sides cut with a knife, fish on the plate with *Paeoniae Radix Alba* juice added into the pot and steamed for eight minutes and cooked	Tonifying qi, nourishing the kidneys, detoxifying and avoiding epidemics	*Home remedies* [127]
Meigui Moyu decoction	Rose petals 30 g, *Paeoniae Radix Alba* (Baishao) 15 g, Cuttlefish 200 g	Rose and *Paeoniae Radix Alba* in gauze, cuttlefish washed and removed and boiled together to make soup	Regulating qi, dispersing liver, relieving depression and nourishing blood	*Aquatic cuisine* [128]
Shaofu Shourou decoction	*Paeoniae Radix Alba* (Baishao), *Cyperus rotundus* L. (Xiangfu), *Citrus aurantium* L. (Zhiqiao) 6 g each, 100 g lean pork, seasoning in moderation	First of all, wash the pork lean meat, shredded, sizing, the remaining drugs with water decoction to make juice, added into the pork lean meat and cooked; seasoned food, 1 dose per day	Tonifying the liver and regulating qi, strengthening the spleen and harmonizing the stomach	*Dietary therapy for gastrointestinal disorders* [129]

## Data Availability

The data presented in this study are available in article.

## Abbreviations

PRA: Paeoniae Radix Alba; TGP, total glucosides of paeony; PF, paeoniflorin; GCAFs, gastric cancer-associated fibroblasts; EMT, epithelial–mesenchymal transition.
